# The current status of IVF: are we putting the needs of the individual first?

**DOI:** 10.1016/j.eclinm.2023.102343

**Published:** 2023-11-23

**Authors:** eClinicalMedicine

In Oldham General Hospital, Oldham, UK, at 11:47 pm on July 25, 1978, the world of fertility medicine changed forever with the birth of 5 lb 12 oz Louise Joy Brown, the first baby to be born after successful in vitro fertilisation (IVF). Since that Tuesday night 45 years ago, the International Committee for Monitoring Assisted Reproductive Technologies estimates that at least 12 million babies have been born as a result of IVF and other assisted reproductive technologies (ARTs).

Most of the available data point to year-on-year increases in the number of IVF cycles. In the UK, the Human Fertilisation and Embryology Authority (HFEA) reported that 55,000 women had around 76,000 fresh and frozen embryo transfer IVF cycles in 2021, an increase of 2000 individuals since 2019. In Australia, there was a 6.2% increase in the number of IVF cycles initiated from 2018 to 2019, and in Japan, 498,140 IVF cycles, the highest number on record, were reported in 2021, up from a steady average of around 450,000 cycles yearly since 2016.

Although IVF success rates vary based on a number of factors, including age, lifestyle factors (such as smoking status and alcohol consumption), and oocyte and sperm quality, the overall success rate of IVF procedures has increased from 6% in the 1990s to 27% in 2021 in the UK. This improvement is the result of new technological advances and constant work to update, refine, and optimise IVF procedures and regimens. However, with the improvements in ARTs and increased uptake of IVF treatments, a series of expensive IVF add-on treatments—optional, non-essential, procedures and treatments that are used alongside ARTs to potentially improve chances of pregnancy—have become commonplace, often with little evidence supporting their efficacy.

In October, 2023, HFEA updated their traffic light grading system for IVF add-ons. The grades ranged from “being effective at improving the treatment outcome” (green) to showing “potential safety concerns or reduced effectiveness” (red). Of the 13 treatment add-ons assessed, none were rated as green overall, with four graded red due to a lack of supporting data or safety concerns. Endometrial receptivity testing, during which gene expression of an endometrial wall biopsy sample is assessed to predict the best time for embryo transfer, is a key example of a procedure rated as red by HFEA due to a lack of supporting evidence, with only one randomised trial to have assessed the procedure, which provided inconclusive evidence of its effect. Until strong evidence in favour of receptivity testing is reported, the process—which can cause cramping and is associated with a risk of bleeding and infection—should not be offered. Immunological therapies in healthy women was rated red due to severe safety concerns. The use of steroids, intravenous immunoglobulins, or TNF blockers has been suggested to control uterine natural killer cells, which some have thought might affect chances of pregnancy. HFEA reports that there is no evidence to suggest that immune system cells can prevent a pregnancy in healthy women; crucially, the regimens can result in severe allergic reactions and infections that can affect the baby. However, a nuanced and personalised approach is needed because for women with immunological diseases, these therapies can be vital. Pre-implantation genetic testing for aneuploidy (PGT-A) is another key example of the balancing act clinicians face. PGT-A identifies the most viable embryos for transfer by screening for aneuploidy; therefore, the process intrinsically reduces the number of embryos available for transfer in most women, for which HFEA graded the process red. However, HFEA rated PGT-A green for reducing the risk of miscarriage, particularly in older women and women with a history of miscarriage. The recommendations raise the question, should PGT-A be avoided to increase the number of embryos that could be transferred, or should the process become an integral part of IVF to minimise the risk of and psychological harms caused by miscarriage? Ultimately, a balance must be struck with the specific needs of each individual at its core.

In the UK, NICE recommends that three rounds of IVF be included on the NHS; however, some local integrated care boards limit this to just one cycle, adding more pressure for the immediate success of IVF. This leads many to the private sector where the cost per cycle averages between £3000 and 4000, but can spiral to close to £9000 per cycle following the addition of the add-ons and extra fees. As with the postcode lottery for NHS IVF treatment, an individual’s address can impact the amount they pay for private IVF care, with people in London paying £2000–3000 more than those in other parts of the country. In the USA, the average price of one IVF cycle ranges from US$12,000 to 17,000, increasing to $25,000 if medications are needed, and with insurance coverage dictated at a state—not a federal—level only 14 states mandate that IVF be included in insurance plans. Similarly, in Japan, many IVF treatments are not covered by the national insurance scheme, with costs starting at ¥300,000–500,000 and increasing with each add-on used.

WHO estimates that one in six people are affected by infertility. The increase in uptake and growing importance of IVF to so many people across the globe places ever greater scrutiny on the field. The updated HFEA guidelines are a welcome update, but they also represent the tightrope on which fertility specialists balance. For many, IVF represents a last chance for a biological child of their own. The field of fertility medicine must be encouraged to continue to innovate, invent, and assess the process of IVF to ensure the best outcome and experience possible for each individual, but it is clear that this cannot be funded by potential parents paying for the use of unnecessary, and in some cases potentially harmful, IVF add-ons, which might not increase their chance of success. The individuals who use IVF should be at the heart of every decision made. The patient, not their purse, should always be the focus.

